# Amalgam and Kindred Poisons

**Published:** 1896-05

**Authors:** Henry Sheffield

**Affiliations:** Nashville, Tenn.


### AMALGAM AND KINDRED POISONS.

HENRY SHEFFIELD, M. D., NASHVILLE, TENN.

Health is a subject in which every individual is interested. How to secure it, how to protect it from impairment by poisonous substances and other detrimental agents, gives man the most anxious thoughts of life, and the philanthropist his most benevolent study.

Filling carious teeth with amalgam is destructive to health—it, with other poisonous substances, will be the theme of this article.

The poisonous effects of amalgam are not generally recognized, are not cared for properly, therefore its injurious effects should be as widely known as its use.

One of the ingredients of amalgam is quicksilver, which will rapidly oxidize when exposed to the air. Those men who mine it and inhale its vapors are salivated; they suffer from cerebral diseases; they are palsied; they sink into marasmus and die prematurely.

Plants that are exposed to the vapor of quicksilver, in a close room, will perish in a few days.

It is but a few years since a merchant ship left San Francisco, and in her cargo was a large quantity of quicksilver. It was not securely confined; it escaped and ran into the hold of the ship. There it came in contact with bilge water, by which it was oxidized, and its vapor soon permeated the hull of the ship. The crew of the ship were salivated, the canary bird caged in the cabin, the pigs and the fowls, each in their own quarters, and the rats in the hold, died from inhaling the poisonous vapor.

When quicksilver is dissolved in Nitric Acid, the product is Nitrate of Mercury. Let me enumerate some of the poisonous effects of Mercury on the human family. A metallic taste in the mouth, headache, soreness and sponginess of the gums, pain in the sockets of the teeth when pressed together, fetid breath, ptyalism, ulceration of the mucous membrane of the mouth, fauces, larynx, and bronchia, loss of voice, hectic fever, profuse perspiration, emaciation, and death.

Quicksilver will dissolve in strong acid, it will oxidize in open air, in contact with fetid water, in the mouth when exposed to acids and vitiated secretions, and when swallowed with them is absorbed by the stomach, and its effects poisonous. The quantity of this oxide taken into the stomach in one week, or a month, will be comparatively small, but when swallowed, as it is, for years, will certainly produce the poisonous effects of Mercury, and in nearly the same order as heretofore enumerated.

The mercurial effect of this oxide is not as rapidly and extensively developed in persons of bilious, phlegmatic temperaments as in persons of nervous sanguine temperaments, who are by heredity predisposed to glandular and bronchial diseases.

We know that great grief, fright, intense anger, may so poison the milk of the nursing mother as to carry death to the child. Professor Elmer Gates, of the Smithsonian Institute, has not only isolated the poison and shown it in crystals, but has demonstrated that bad and unpleasant feelings create harmful chemical products in the body which are physically injurious, while good, pleasant, and benevolent feelings create beneficial chemical products, and these products may be detected by chemical analysis in the perspiration and urine.

Now, if mental emotions can create poisonous chemical products, it is absolutely certain that chemical substances introduced into the blood will destroy its purity and create disease in some part or organ of the body for which that substance has an elective affinity.

A great many persons possess an idiosyncrasy peculiar to themselves. One person will faint from one odor, another from a different one. One has fainted from the odor of copper, while hundreds of others have worn it in contact with the body, and it has relieved them of cramp, or rheumatism, or neuralgia.

These persons who have been salivated are particularly sensitive to Mercury; in them I have frequently seen ulcers of the mouth and fauces produced by a few doses of Mercury, each dose containing less than the one-thousandth part of a grain. Those persons who have been salivated and have amalgam fillings in their teeth, frequently suffer from mercurial rheumatism and other symptoms of that poison; in fact, some are walking barometers, and by their pain can foretell a change of weather.

When the liver has been stored with atoms of Mercury, it can be stimulated to increased secretion by acids, hence their frequent use.

The proverb, "The fathers have eaten grapes and the children's teeth are set on edge," is an eternal fact which appears to be little known and less observed in this country. The effects of Mercury and other poisonous substances are not confined to parents who use them, but are transmitted to their children, "even to the third and fourth generations."

It is not, therefore, what comes out of the parents' mouths that defiles them and produces disease in their children, but that which they put into them. The use of alcoholic drinks, drugs, tobacco, Morphia, with many other poisons, is destroying the physical stamina and mental equilibrium of native Americans and making them selfish and evil. The free use of deleterious substances will degrade the natural body and make it an unfit temple for the soul.

If you would learn the effects of poisonous substances, examine carefully the children of native Americans who have used such enormous quantities of them. You will find them with enlarged tonsils, glandular swellings, skin diseases, catarrhal affections of the eyes, ears, nose and throat, subject to croup, teeth and bones imperfectly developed; they easily succumb to inflammatory conditions or marasmus ends their early career. Our asylums for the vicious, the insane, the orphan, the blind, the mute, the deaf are crowded with them.

These facts show the rapid decline of native Americans, physically, mentally, and morally during the past century, and if the same course is pursued of constantly swallowing poisons, it will not require a prophet to foretell their ultimate extinction.

"Weighed in the balance and found wanting" is the writing on the wall; and unless philanthropists and reformers waken native Americans to their danger, they will not escape their doom.

For forty years patients have come to me with teeth filled with amalgam, each one suffering with some of the symptoms enumerated in poisoning by Mercury. After the amalgam had been removed, and their teeth refilled with gold, they soon began to improve and ultimately recovered, except those who had ulceration of epiglottis, larynx, trachea, and bronchia, accompanied with hectic.

The facts presented with my own statement *are positive evidence*, and it cannot be lessened or destroyed by any amount of *negative evidence* nor ridicule.

To my patients I state plainly the effect of amalgam fillings and its disastrous consequences. They then consult their favorite dentist, who declares their teeth to be in good condition, and he is acquainted with persons who have worn amalgam fillings for many years without any injury, and he knows it can do them no harm.

To this statement I will reply, that he cannot know (only believes) that any one person can resist the poisonous effects of any substance, or perform any act whatsoever, because another person has done so successfully. For example: Dr. Winslow, of Boston, could lift a dozen men at the same time, therefore my patient can do the same thing. Professor Sandow can do the somersault with fifty pounds in each hand, therefore my patient can do the same thing. Such deductions are false, misleading, and harmful.

Every individual is entitled to his own opinion and belief, in the defense of which he will often risk his own life. That does not prove his belief to be either true or false; only shows his own firmness and fortitude.

If it is founded on falsehood or superstition it is worthless to others who know the facts.

Now, it is utterly impossible to have a clear brain, with virtuous, benevolent, and honest impulses, surmounting a diseased and depraved body. We, therefore, sadly need school-houses where able preceptors can teach new women and children "the truth, the whole truth, and nothing but the truth" about physiology, toxicology, hygiene, and every other subject that has any bearing on health ; how to protect and perpetuate it, and how to transmit it to our children.

Health is a universal need, as that only can modify avarice, lust, and crime, and ultimately overcome them.

When native Americans all become sound in body and brain, then, and then only, can they closely follow the examples of Christ ; then, and then only, will they practice religion in its purity; then, and then only, will justice, harmony, peace and prosperity prevail among them.—*The Homœopathic News.*

